# Bleomycin-induced pneumonitis in a young Ghanaian male with Hodgkin's Lymphoma

**DOI:** 10.4314/gmj.v54i4.12

**Published:** 2020-12

**Authors:** Yvonne A Dei-Adomakoh, Jane S Afriyie-Mensah, Hafisatu Gbadamosi

**Affiliations:** 1 Department of Haematology, University of Ghana Medical School, College of Health Sciences University of Ghana, Accra, Ghana; 2 Department of Medicine and therapeutics, University of Ghana Medical School, College of Health Sciences, University of Ghana, Accra, Ghana; 3 Department of Radiology, Korle Bu Teaching Hospital, Korle Bu, Accra, Ghana

**Keywords:** bleomycin, pneumonitis, Ghana, Hodgkin's lymphoma

## Abstract

We report a case of a young Ghanaian male who developed Bleomycin Induced Pneumonitis (BIP) after being treated for Hodgkin's Lymphoma. Pulmonary toxicity is the most feared complication of bleomycin therapy despite its effectiveness in achieving cure in patients with Hodgkin's lymphoma and germ cell tumors. BIP has a significant mortality rate if detected late and a high index of suspicion is required in all patients on bleomycin-based therapies with sudden onset of respiratory symptoms.

## Introduction

Pulmonary toxicity is the most serious adverse effect of bleomycin therapy despite its effectiveness in achieving cure in patients with Hodgkin's lymphoma and germ cell tumors.^1^ Its presentation can be severe and life-threatening occurring in approximately 10% of patients receiving bleomycin therapy with 10–20% mortality.^2^ It forms one of the vital components of a combination therapy including adriamycin, vincristine and dacarbazine (ABVD). We present this case focusing on the clinical presentation, differential diagnoses, management and prognosis of bleomycin induced pulmonary damage, aiming to increase its awareness and promote early recognition of the condition among at risk patients.

## Case Report

A young male was diagnosed with histology confirmed Hodgkin's lymphoma (mixed cellularity type) stage 2A at age 14 when he presented with left discrete cervical and supraclavicular lymphadenopathy in the year 2007. He was then treated with 6 cycles of COPP (Cyclophosphamide, Oncovin, Prednisolone and Procarbazine). As a result of persistent cervical lymphadenopathy after chemotherapy, he further had sessions of localized radiotherapy (40Gy full mantle field) resulting in clinical resolution of the disease.

**Figure 1:**
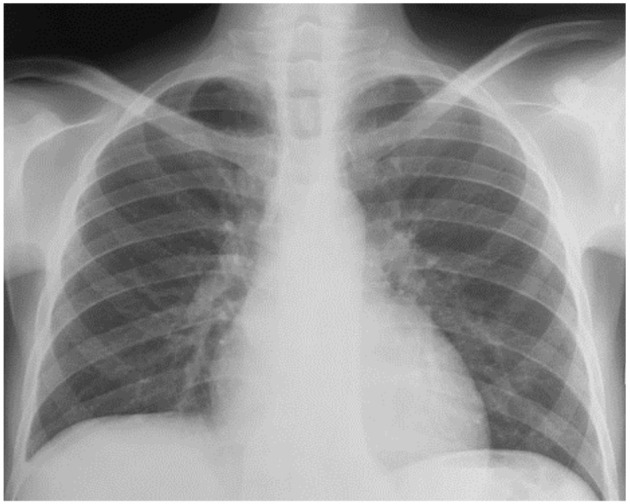
Normal initial Chest radiograph

He remained in clinical remission for about 10 years until December 2016 when he presented with B symptoms (fever, night sweats and weight loss), anaemia (Hb 4.6g/dl), jaundice, bipedal oedema, hepato-splenomegaly and multiple retroperitoneal lymph nodes on abdominal ultrasound. Having a raised lactate dehydrogenase (LDH) (778U/L), he was diagnosed with relapsed staged 4 HL. His chest X-ray (CXR) was normal (Figure 1). Treatment was initiated using ABVD (day 1 and 14 cycles) with significant clinical improvement (weight gain, raised Hb (8.7g/dl) and normalization of LDH after 5 cycles of chemotherapy).

Patient however defaulted and unfortunately returned to clinic 10 months later with anaemia (Hb 5.9g/dl) and systemic symptoms as above, suggestive of progressive HL disease. He received 12 cycles of ABVD without fail and again had a good response (Hb 11.2g/dl).

He presented acutely a month later with a dry cough that began just after the 10^th^ dose of chemotherapy that had progressively worsened. He had associated dyspnea on exertion. He was tachypnoeic (RR-68cycles/min), afebrile (36.9), tachycardic (HR 134beats/min), SpO2 of 68–75% on room air (increased to 85–90% on 12–15l/min oxygen via a non-rebreather mask).

**Figure 2:**
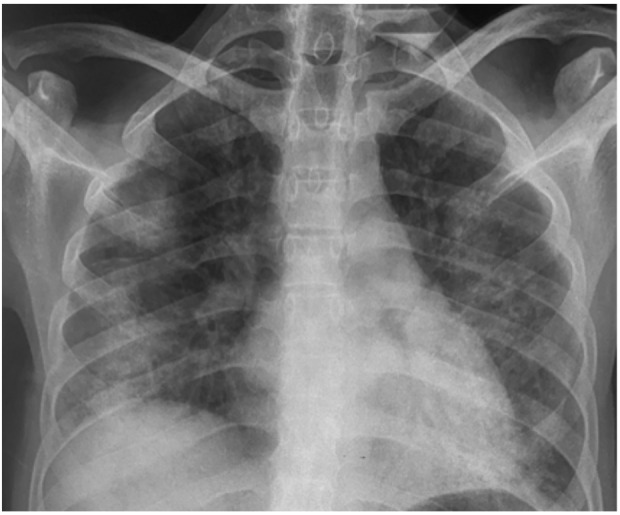
Chest radiograph showing Ground glass opacification in both middle and lower lungs zone

He had no peripheral lymphadenopathy, but reduced breath sounds with course crepitations in both mid-lower lung zones. He was managed as bilateral pneumonia secondary to chemotherapy-induced immunosuppression with IV ceftriaxone and azithromycin. His Hb was 11.6g/dl, WBC-17.1 × 10^9^/l with a neutrophilia, ESR-48mmfall/hr, CRP-18mg/ml and elevated LDH-1000U/L. His CXR showed bilateral ground glass opacification (GGO) in the mid to lower lung zones with no pleural effusion (Figure 2). Differentials of possible atypical pneumonia, *Pneumocystis jiroveci* pneumonia (PJP), disseminated Koch's disease and progression of HL with lung involvement were made. Scanty sputum obtained for culture, fungal elements and geneXpert were negative. Bronchoscopy to obtain bronchoalveolar lavage (BAL) for detection of *Pneumocystis jiroveci* was not performed due to the pertaining severe hypoxia but empirically started on high dose cotrimoxazole and prednisolone by day 2 of admission. Patients' clinical picture after 7 days of IV antibiotics, high dose cotrimaxazole and prednisolone was static and transfer to ICU for possible mechanical ventilation was declined by relations.

**Figure 3:**
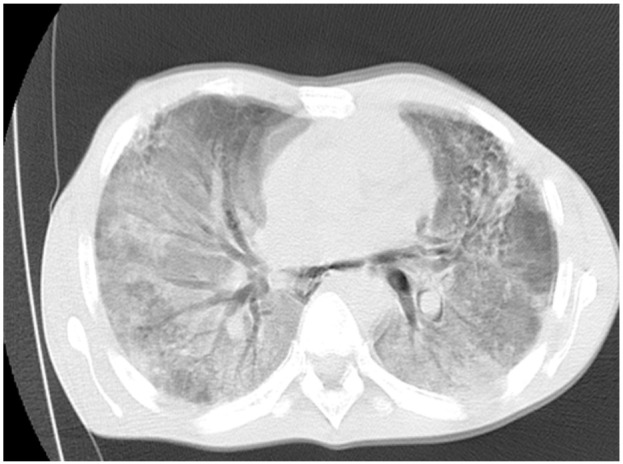
HRCT, lung window: worsening bilateral ground glass opacification with associated reticulations

**Figure 4:**
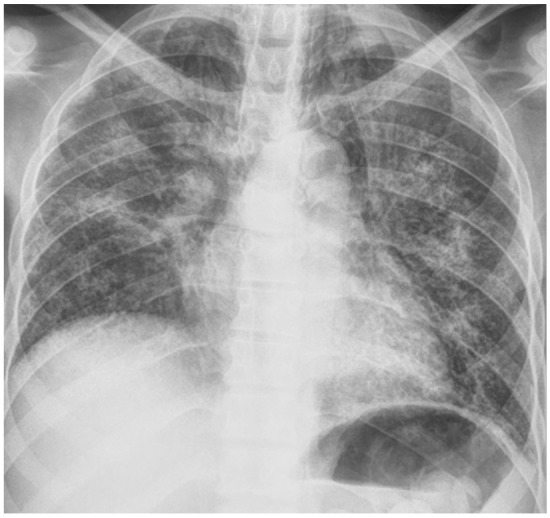
Follow-up chest radiograph – coarse bilateral patchy lung opacities with progression of reticular opacities

High-Resolution Chest CT (HRCT) on day 4 showed extensive bilateral GGOs with air bronchograms and areas of alveolar infiltrates as well as some background reticulations (suggestive of fibrotic changes) with relatively spared lung apices (Figure 3). Based on the clinical picture and HRCT findings, a diagnosis of probable bleomycin induced pneumonitis (BIP) with a differential of PJP was made. Patient completed 21 days of PJP therapy, but high dose prednisolone was continued on account of the diagnosis of probable BIP. A follow-up chest radiograph done on day 20 showed progression of the fibrotic changes (Figure 4).

After 4 weeks of high dose prednisolone, patient made a modest improvement with reduction in oxygen requirement (5L/min via a simple face mask to keep SpO2 at 88–90%).

Spirometry showed an FVC of 0.87L (19%), FEV1 of 0.72L (19%) and an FEV1/FVC ratio of 83 suggestive of very severe restrictive defect. Patient was discharged on long-term oxygen via simple face mask with a plan to systematically taper dose of prednisolone. Over the following four months, he progressively got dyspnoeic with increasing oxygen demands.

Repeated CXR showed progressive bilateral opacifications (Fig 4). He developed secondary polycythaemia from persistent hypoxia (Hb-17.1g/dl; hematocrit-54%) and LDH was 724 U/L. Based on the absence of fever, lack of response to treatment for PJP, severe restrictive lung defect, chest image findings and respiratory deterioration over a 6-month period, the diagnosis of Bleomycin-induced pneumonitis with progression to lung fibrosis and hypoxaemic respiratory failure was secured. Patient was reported to have died in his sleep barely five months after the acute presentation.

## Discussion

Bleomycin has long been known for its fibrogenic and anti-neoplastic properties.^3^ Its use in our setting has been for > 30 years but there has been no documented case of BIP to the best of our knowledge. Although an antibiotic of a kind, its role in medicine has mainly been as an anticancer agent and a sclerosant for pleurodesis with significant efficacy.^3^,^4^ In the field of research it is used to induce lung fibrosis in mice modules to enhance understanding of disease pathogenesis and the development of drug therapy.^5^ Despite this adverse effect (the lung being most susceptible), it forms an integral component of a combination of chemotherapeutic agents used in the management of lymphoma and germ cell tumors as well as Kaposi sarcoma, cervical cancer and squamous cell carcinoma of the head and neck.^2^ The prevalence of bleomycin lung injury among adults receiving ABVD for HL is much higher ranging between 10–53% with an estimated mortality rate of 4–5%.^6^

Histological pattern of bleomycin lung injury varies including organizing pneumonia, hypersensitivity pneumonitis and interstitial pneumonitis with the latter being the commonest. 2,7 Proposed mechanism of lung injury is the production of free radicals through DNA strand scissoring resulting in alveolar epithelium and basement membrane injury subsequent to pneumonitis and interstitial fibrosis later.^8^ Radiologically, GGOs, reticular opacities and consolidation are commonly seen on chest radiograph with subpleural and lower lung zone predilection.^9^

Moderate to severe disease tend to be diffuse with involvement of the mid and upper lung zones as evident in our case report. Chest radiographs due to their low sensitivity may miss early findings of the disease making HRCT the gold standard which exhibits significant correlation with underlying histopathology.^9^ Due to the varied histological spectrum, HRCT appearances may also vary but classic diffuse bilateral ground glass opacities, alveolar infiltrates and reticular markings as presented here is consistent with a pattern of interstitial pneumonitis with progression to irreversible lung fibrosis.^8^

Symptoms of BIP may be sub-acute typically occurring during treatment (averagely 1.2–8.2 months of therapy initiation) but could present months after completion of chemotherapy.^10^,^11^ Similarly, our case appeared to have developed a dry cough and breathlessness during treatment, but his symptoms unfortunately went unreported and further had two more cycles, shortly after which he progressed rapidly into acute respiratory failure.

The clinical presentation and radiological pattern of BIP is not unique being similar to many other respiratory infections and malignancies. The diagnosis is therefore secured by exclusion of other possible causes and a high index of suspicion in at-risk patients. Infective pneumonias, particularly those caused by opportunistic viral and fungal organisms, are important considerations as patients are likely immunosuppressed from chemotherapy.^12^ With poor microbial yield on blood and sputum cultures, empirical treatment for the most likely infective causes are typically initiated. Similar some reported cases of BIP, we unsuccessfully treated our patient for community acquired pneumonia, suspicion based on the clinical presentation and leukocytosis.^13^,^14^

Leukocytosis and a rise in non-specific acute phase reactants such as ESR and CRP also occur in acute inflammatory lung conditions including BIP making it more difficult to distinguish infective lung inflammation from a non-infective one.^15^ Serum procalcitonin assay have been shown to be a sensitive biomarker which helps one make this distinction.^16^ *Pneumocystis jiroveci* pneumonia (PJP) although more common in non-Hodgkin's lymphoma (NHL) than HL, was considered likely in this patient judging from the clinical and radiological similarities it shares with BIP.^9^,° Empirical treatment for PJP is encouraged in such immunosuppressed patients while awaiting confirmatory tests especially where pneumocystis silver stains of BAL or sputum can remain positive10 -14 days after treatment initiation.^13^,^17^,^18^ Elevated serum LDH levels also occur in PJP infections and could be an important marker of the disease in HIV infected individuals but less so in non-HIV immunosuppressed cases where the underlying malignancy could drive the increased levels of LDH making interpretation unclear as seen in our case report.^19^ LDH however is a non-specific indicator of organ damage and may be elevated in variety of lung diseases/infections.^19^

Although evidence suggests that doses of bleomycin > 400U (400,000IU) and patients aged over 40 are at an increased risk of BIP, our patient was younger (26years) and had lesser dose given. 8,20 Our patient received repeated doses of bleomycin containing regimen within an 18month period due to relapses in background advanced HL. These repeated exposures over the short period, totaling 274,000IU, could have been the identifiable risk factor here.

Prognosis in BIP cases with respiratory failure requiring ICU care appears to be guarded especially in those with onset of lung fibrosis as noted in our patient.^21^ Good response in 50–70% of BIP cases treated with high dose prednisolone has been observed resulting in clinical and radiological resolution of the disease.^22^,^23^ This response is much enhanced in those with the pattern of organizing pneumonia or hypersensitivity pneumonitis.^10^ A high steroid dose of 0.75–1mg/kg is initially recommended with dose tapering after 4–8weeks based on patient symptoms with no consensus on treatment duration.^22^ In our case, the poor response to steroids could be attributed to the late recognition of the symptoms which led to continuous exposure to bleomycin with lung fibrosis even at the time of presentation. Early disease detection and immediate withdrawal of therapy as proposed by the National Comprehensive Cancer Network, has been the most appropriate management strategy with good prognosis as this prevents progression of acute inflammatory changes to irreversible lung damage.^24^

Although the NCCN recommends pulmonary Function tests (PFTs) and CXR prior to bleomycin therapy initiation, this is not routinely done in our clinical practice. For asymptomatic patients, significant reduction in pulmonary function tests especially DLco offers a clue for early disease detection.^25^ There are however, controversies regarding serial PFTs and CXR during treatment with no clear guiding protocol on how often this should be done. Some studies have however, that reliability on reduction in lung function parameters alone to predict bleomycin lung damage could be misleading resulting in unnecessary cessation of effective bleomycin therapy in some patients.^26^ Yet without any screening mechanism, one wonders how many cases are missed and perhaps treated unsuccessfully as bilateral pneumonia. Though a great interventional measure recommended by NCCN, the cost implications of serial PFTS and CXR could be huge in most health facilities. Hence, aside the initial screening PFTs and CXR, a comprehensive medical assessment prior to the beginning of each cycle, designed to actively extract symptoms, as well as educate patients on typical symptoms of BIP could be quite useful in low resource settings.

Further investigations such as PFTS and chest imaging will only be requested based on outcomes of such screening tools.

## Conclusion

Early detection of BIP can be lifesaving, hence all patients on bleomycin-based therapies should have baseline CXR and PFTs. Active, not passive search for respiratory symptoms prior to every cycle is key to preventing respiratory impairment and mortality.
